# Association of Chinese herbal medicine use with the depression risk among the long-term breast cancer survivors: A longitudinal follow-up study

**DOI:** 10.3389/fpsyg.2022.884337

**Published:** 2022-08-18

**Authors:** Shu-Yi Yang, Hanoch Livneh, Jing-Siang Jhang, Shu-Wen Yen, Hua-Lung Huang, Michael W. Y. Chan, Ming-Chi Lu, Chia-Chou Yeh, Chang-Kuo Wei, Tzung-Yi Tsai

**Affiliations:** ^1^Department of Chinese Medicine, Dalin Tzuchi Hospital, The Buddhist Tzuchi Medical Foundation, Chiayi, Taiwan; ^2^Department of Biomedical Sciences, National Chung Cheng University, Chiayi, Taiwan; ^3^Rehabilitation Counseling Program, Portland State University, Portland, OR, United States; ^4^Department of Rehabilitation, Dalin Tzuchi Hospital, The Buddhist Tzuchi Medical Foundation, Chiayi, Taiwan; ^5^Epigenomics and Human Diseases Research Center, National Chung Cheng University, Chiayi, Taiwan; ^6^Division of Allergy, Immunology and Rheumatology, Dalin Tzuchi Hospital, The Buddhist Tzuchi Medical Foundation, Chiayi, Taiwan; ^7^School of Medicine, Tzu Chi University, Hualien, Taiwan; ^8^School of Post-Baccalaureate Chinese Medicine, Tzu Chi University, Hualien, Taiwan; ^9^Department of General Surgery, Dalin Tzuchi Hospital, The Buddhist Tzuchi Medical Foundation, Chiayi, Taiwan; ^10^Department of Environmental and Occupational Health, College of Medicine, National Cheng Kung University, Tainan, Taiwan; ^11^Department of Nursing, Tzu Chi University of Science and Technology, Hualien, Taiwan; ^12^Department of Medical Research, Dalin Tzuchi Hospital, The Buddhist Tzuchi Medical Foundation, Chiayi, Taiwan

**Keywords:** breast cancer, Chinese herbal medicines, depression, cohort study, risk

## Abstract

**Background:**

Breast cancer patients are at elevated risk of depression during treatment, thus provoking the chance of poor clinical outcomes. This retrospective cohort study aimed to investigate whether integrating Chinese herbal medicines citation(CHM) into conventional cancer therapy could decrease the risk of depression in the long-term breast cancer survivors.

**Methods:**

A cohort of patients aged 20–70 years and with newly diagnosed breast cancer during 2000–2008 was identified from a nationwide claims database. In this study, we focused solely on survivors of breast cancer at least1 year after diagnosis. After one-to-one matching for age, sex, and baseline comorbidities, breast cancer patients who received (*n* = 1,450) and did not receive (*n* = 1,450) CHM treatment were enrolled. The incidence rate and hazard ratio citation(HR) for depression between the two groups was estimated at the end of 2012. A Cox proportional hazard model was constructed to examine the impact of the CHM use on the risk of depression.

**Results:**

During the study period, the incidence rate of depression was significantly lower in the treated cohort than in the untreated cohort [8.57 compared with 11.01 per 1,000 person-years citation(PYs)], and the adjusted HR remained significant at 0.74 (95% CI 0.58–0.94) in a Cox proportional hazards regression model. The corresponding risk further decreasing to 43% among those using CHM for more than 1 year.

**Conclusion:**

Finding from this investigation indicated that the lower risk of depression observed in breast cancer patients treated with CHM, suggesting that CHM treatment should be considered for disease management toward breast cancer. Yet, the optimal administered dose should be determined in further clinical trials.

## Introduction

Depression is a chronic medical condition that impacts the cognitive, affective and physical wellbeing of a person (Cui, 2015). Globally, depression affects approximately 350 million people (Cross et al., 2014), and is particularly common among people with chronic and life-threatening diseases, especially in patients affected by cancer (Delgado-Guay et al., 2009). Recently, the advances in treatment modalities have improved the survival for cancer survivors; nevertheless, the higher survival rate does not represent that they have successfully adapted or positively coped with the cancer. In contrast, the profound functional and visible changes resulting from such treatments, including surgery, chemotherapy, and radiation therapy, can insidiously lead to the onset of psychiatric disorders throughout the cancer experience (Chan et al., 2015).

Breast cancer is the most common form of malignancy in women, and affected patients are at a higher likelihood of experiencing depression, a condition aggravated by the necessity to undergo continuous life-saving treatments. After administering their cancer-related questionnaire to 179 women with breast cancer, Kamińska et al. (2015) found that breast cancer surgeries contributed to a higher prevalence of depression, reaching 64% for women who underwent mastectomy and 52.4% for women who had breast conserving treatment. Additionally, one recent meta-analysis of 18 studies, representing different countries, disclosed that estimated depression prevalence among these women ranged from 14 to 95.9%, with a pooled average of 46.83% (Ahmadi Gharaei et al., 2019). Notably, once individuals with breast cancer develop concomitant depression, their medical costs increased by nearly 200% (Mausbach et al., 2018). Worse yet, further aggravating these findings, their likelihood of mortality compared to patients with cancer alone was reported to more than double (Chan et al., 2015). These alarming data suggest that interventions geared toward minimizing the likelihood of depression are of utmost importance during cancer therapy.

Chinese herbal medicine citation(CHM) has shown promising results in improving the body’s ability to fight malignant disease. Several of these herbs were further proven to improve cognitive function through the suppression of monoamine oxidase activity and oxidative stress as well as the modulation of the hypothalamic-pituitary-adrenal axis activation (Zhang et al., 2018; Ahmadi Gharaei et al., 2019). Take use of Shao-Yao-Gan-Cao-Tang as an example, it has been found to have a beneficial effect on reducing emotional distress owing to its ingredients of Paeonia lactiflora Pall, and Glycyrrhiza uralensis (Liu C. T. et al., 2017; Chen et al., 2018). The latter ingredient even was proved to exert antidepressant effects on depressive rat model *via* regulating HPA Axis and the level of amino acids neurotransmitters (Wang et al., 2008). In this context, these biological activities of CHM may be of value for managing depression in patients with breast cancer. As of now, to the best of our knowledge, no study has so far investigated the long-term effects of CHM on depression prevention in breast cancer patients. To address this issue, we used administrative data to create an objective measure to determine if adding CHM into the conventional therapy could lower the incidence of depression among breast cancer patients.

## Materials and methods

### Data source and identification of patients with breast cancer

Taiwan’s NHI program, launched in 1995, is a single-payer government-operated compulsory health insurance program. As of 2015, up to 99.6% of Taiwan’s population was enrolled in the NHI program Research data for this retrospective cohort study were obtained from the Longitudinal Health Insurance Database citation(LHID) administered by the Ministry of Health and Welfare. The LHID contains data on one million beneficiaries randomly sampled from the Registry for Beneficiaries of the NHI Research Database. The use of multistage stratified systematic sampling ensures no significant deviations in the distribution of sex and age between the LHID enrollees and the general population (The National Health Research Institutes, 2002). The claim files include inpatient and outpatient demographics, primary and secondary diagnoses, procedures, prescriptions, and medical expenditures. The Ministry of Health and Welfare manages the claims data and provides scrambled random identification numbers for insured patients to protect their privacy. Accordingly, the Institutional Review Board and Ethics Committee of Buddhist Dalin Tzu Chi Hospital approved this study and waived informed consents for all the enrollees (No. B10004021-2).

**FIGURE 1:**
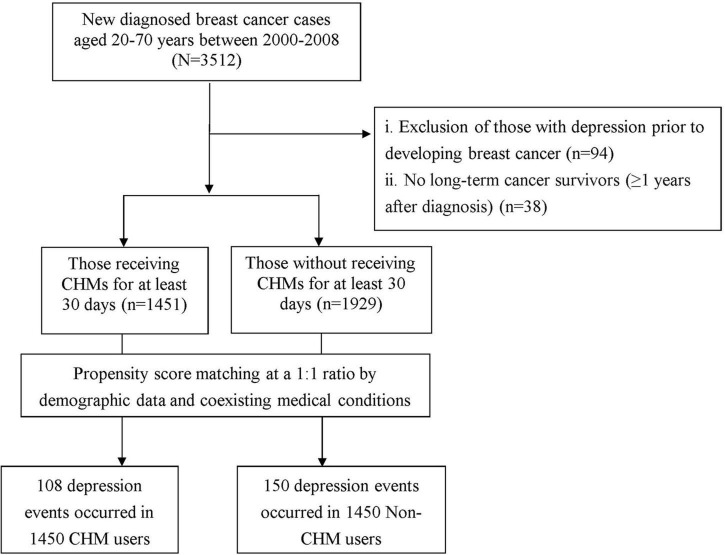
Flow chart of selection and follow-up of study subjects.

In the present study, we used the ambulatory and inpatient data of LHID from 2000 to 2008 to identify subjects with breast cancer and being free of depression diagnosis at baseline. We used the International Classification of Diseases-Ninth Revision-Clinical Modification (ICD-9-CM) code for the identification of patients with breast cancer. Patients aged 20–70 years who were newly diagnosed with breast cancer (ICD-9-CM code: 174) were considered for enrollment in the study. To ensure the identification of new breast cancer cases, we employed a 4-year washout period (1996–1999) to exclude any patients who had been diagnosed with breast cancer before entering the cohort (*n* = 3,512). Meanwhile, to reduce the potential for disease misclassification, only those with catastrophic illness certification due to breast cancer were recruited. In Taiwan, insured persons with major chronic conditions can apply for catastrophic illness certification that grants exemption from co-payment. The date on which the patient gained approval for catastrophic illness registration due to breast cancer was considered the index date (Figure 1).

### Outcome of interest

The study outcome was the first diagnosis of depression, which included major depressive disorder (ICD-9-CM codes: 296.2 and 296.3), dysthymic disorder (ICD-9-CM code: 300.4), and depressive disorder not elsewhere classified (ICD-9-CM code: 311). In this study, the diagnosis of depression was determined if patients were treated with ≧ 3 outpatient treatment claims or ≧1 hospitalization due to depression. We then excluded those who had a prior diagnosis of depression before the breast cancer onset (*n* = 94), and those followed up for less than 1 year after diagnosis of breast cancer (*n* = 38). Afterward, the final cohort included 3,380 patients.

### Exposure of Chinese herbal medicines

Under the NHI program in Taiwan, only certified Chinese medicine physicians are allowed to provide CHM treatment. Based on the formerly established methods, CHM users were identified as those who ever received CHM due to breast cancer for more than 30 days, whereas those treated for 30 days or less were considered to be non-CHM users (Tsai et al., 2016; Li et al., 2019). After this algorithm, 1,451 subjects were classified as CHM users. A comparison cohort was randomly selected from the remaining enrollees who did not receive CHM. For each patient receiving CHM, one patient who did not receive CHM was selected *via* one-to-one propensity score matching. The propensity score value, the predicted probability of CHM exposure, was calculated using logistic regression on the basis of patients’ demographics as shown in Table 1, including age, sex, monthly income, residential area, and comorbidities. Every CHM user was randomly matched with a non-CHM user who had the nearest propensity score, where the difference in the score ranged between –0.1 and + 0.1 (Austin, 2011). Furthermore, to consider immortal time bias (Shariff et al., 2008), the index date for the follow-up period was the date of CHM treatment initiation or, for non-CHM users, the date of the initial breast cancer diagnosis. All patients were followed up to the end of 2012 to confirm depression status. The follow-up time, in PY, was determined by calculating the time interval from the index date to the earliest of the following end points: diagnosis of depression, date of withdrawal from the insurance citation(including death), or the date of December 31, 2012.

**TABLE 1:** Demographic data and selected comorbidities of study subjects.

Variables	Total group	Non-CHM users	CHM users	*p*
	*N* = 2,900 (%)	*N* = 1,450 (%)	*N* = 1,450 (%)	
**Age (year)**				0.52
≤50	1,725 (59.5)	854 (58.9)	871 (60.1)	
>50	1,175 (40.5)	596 (41.1)	579 (39.9)	
Mean citation(SD)	48.9 (9.6)	49.1 (9.7)	48.7 (9.6)	0.31
Monthly income				0.16
Low	1,241 (42.8)	617 (42.6)	624 (43.0)	
Median	1,516 (52.3)	772 (53.2)	744 (51.3)	
High	1,43 (4.9)	61 (4.2)	82 (5.7)	
**Residential area**				0.66
Urban	1,944 (67.0)	972 (67.0)	972 (67.0)	
Suburban	450 (15.5)	218 (15.0)	232 (16.0)	
Rural	506 (17.4)	260 (17.9)	246 (17.0)	
**CCI scores**				
Mean citation(*SD*)	5.6 (2.8)	5.4 (2.6)	5.7 (3.0)	0.07

### Definition of covariates

Covariates in the regression model contained the age, sex, insured amount, urbanization level of enrollee’s residential area, former comorbidities, and medication usage. Regarding insured amount, it was calculated from the patients’ average monthly income and thus, also served as an economic index. Insured income amounts were transformed to ordinal variables according to the ≤ New Taiwan Dollar citation(NTD) 17800, NTD 17,881–43,900, and ≥ NTD $43901. As to the urbanization level, it was calculated according to a published scheme that classified 359 communities in Taiwan into seven stratums, with a higher level indicating a higher degree of urbanization. The classification scheme included the population density, proportion of persons with a college-level education or higher, proportion of elderly residents, proportion of agricultural workers, and number of physicians per 100,000 population. In this work, the urbanization degree was grouped into three strata: urban (levels 1–2), suburban (levels 3–4), and rural (levels 5–7) (Liu et al., 2006). The influence of baseline comorbidities was considered according to medical records during the year preceding cohort entry, using the Charlson-Deyo Comorbidity Index (CCI) (Deyo et al., 1992). The sum of these scores was regarded as a continuous variable for the burden of comorbidities, with higher scores indicating a more severe impact of these comorbidities. To avoid duplicate counting, and possible over-adjustment in the regression model, the diagnoses of relevant tumor were excluded from the CCI score.

### Statistical analysis

Data were analyzed using SAS version 9.3 software (SAS Institute Inc., Cary, NC, United States). Distributions of sociodemographic data and comorbidities between the CHM users and non-CHM users were compared by using the chi-square and Student’s *t*-test. Then we conducted a Cox proportional hazards regression analysis to compute the adjusted hazard ratio citation(HR) with 95% confidence interval citation(CI) of risk of depression in association with CHM use, after adjusting for age, sex, insurance premium (an indicator of income), urbanization level, and CCI simultaneously in the model. To test the robustness of the relationship between CHM use and depression risk, all CHM users were divided into two groups based on time of CHM use: (1) 31–365 days, and (2) ≥ 366 days. The Kaplan–Meier method was used to estimate the cumulative risk of depression in each group, and the difference between groups was assessed using the log-rank test. The proportional hazards assumption was examined by plotting the log [–log (survival function)] vs. the log citation(survival time). A *P* < 0.05 was considered statistically significant.

## Results

Data were analyzed for CHM users (*n* = 1,450) and non-CHM users (*n* = 1,450). Demographic data are shown in Table 1. The mean age of the enrollees was 48.9 ± 9.6 years. The majority of participants had monthly income level of NTD 17,881–43,900 (52.3%) and lived in urbanized areas (82.5%). Regarding comorbidities, the mean CCI score was 5.6 (± 2.8). No significant differences were found between two groups in relation to age, monthly income, residential area, or CCI score after PS matching, showing that the baseline characteristics between two groups were comparable.

**FIGURE 2:**
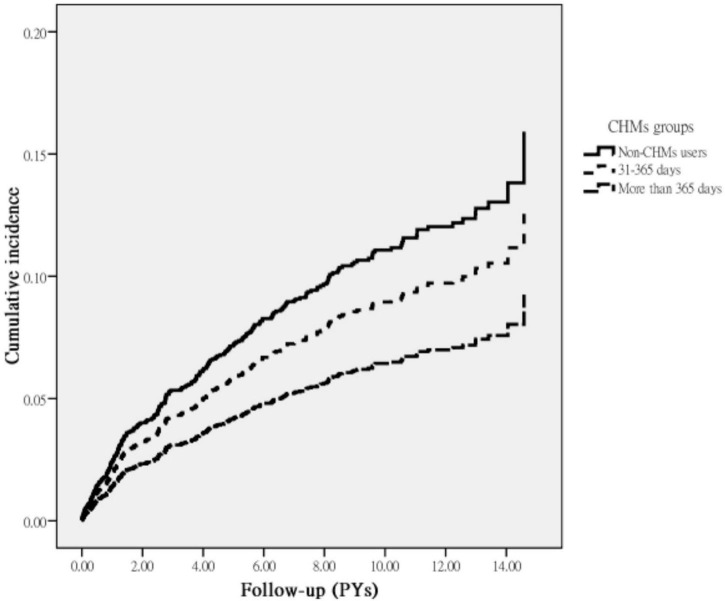
Cumulative incidence of depression in breast cancer patients with and without receiving CHM treatment during the study period (Log-rank test, *P* = 0.02).

In the full cohort, 258 first episodes of depression were identified: 150 in non-CHM users and 108 in CHM users during the follow-up period of 13,629.51 and 12,601.35 person-years citation(PYs), respectively. The incidence of depression was significantly lower among CHM users than non-CHM users (8.57 vs. 11.01 per 1,000 PY, respectively) (adjusted HR = 0.76; 95% CI 0.59–0.96) (Table 2). The use of CHM for more than 1 year was associated with a 43% lower risk of depression. Kaplan–Meier survival curves and log-rank analysis showed a significant difference in survival rate free from depression across three groups during the follow-up period (*P* < 0.01) (Figure 2).

**TABLE 2:** Risk of depression for breast cancer patients with and without CHM use.

Patient group	*N*	Events	PYs	Incidence	Crude HR (95% CI)	Adjusted HR* (95% CI)
Non-CHM users	1,450	150	13629.51	11.01	1	1
CHM users	1,450	108	12601.35	8.57	0.76 (0.59–0.96)	0.74 (0.58–0.94)
CHM use within 31–365 days	1,132	89	9680.75	9.19	0.82 (0.61–1.04)	0.80 (0.61–1.03)
CHM use for more than 365 days	318	19	2920.60	6.50	0.59 (0.36–0.92)	0.57 (0.34–0.92)

*Model adjusted for age, urbanization level, monthly income, and CCI.

**FIGURE 3:**
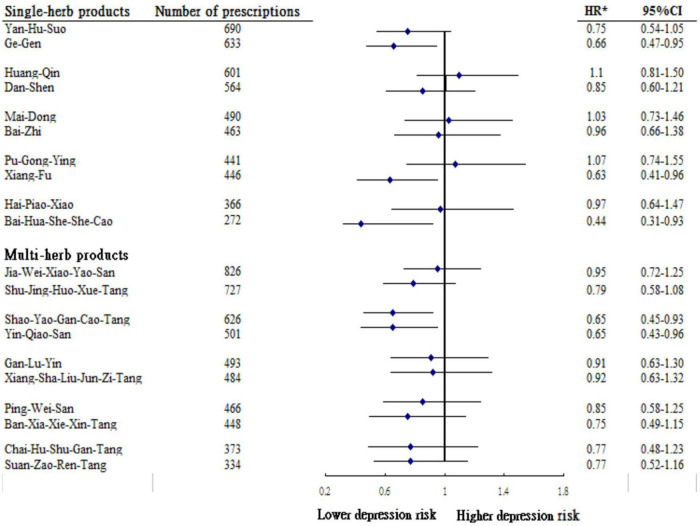
Risk of depression in relation to the top 10 most-used single-herb and multi-herb products among studying participants. *Model adjusted for age, urbanization level, monthly income and CCI.

The most commonly prescribed Chinese herbal products for breast cancer patients are summarized in Table 3. A total of five formulas for the breast cancer treatment were found to substantially lessen the subsequent risk of depression and summarized in Figure 3: Ge Gen citation(*Puerariae Lobatae Radix*), Xiang Fu citation(*Cyperi Rhizoma*), Bai-Hua-She-She-Cao citation(*Hedyotidis Herba*), Shao-Yao-Gan-Cao-Tang citation(*Paeoniae Radix Alba, Glycyrrhizae Radix et Rhizoma*), and Yin-Qiao-San (*Forsythiae Fructus, Lonicerae Japonicae Flos, Platycodonis Radix, Menthae Haplocalycis Herba, Lophatheri Herba, Glycyrrhizae Radix et Rhizoma, Schizonepetae Herba, Sojae Semen Praeparatum, Arctii Fructus*).

**TABLE 3:** Risk of depression in relation to the 10 most-used single-herb and multi-herb CHM products for participants.

Chinese herbal product	Ingredients or generic name	Functional classification
**Single-herb products**		
Yan-Hu-Suo	Rhizoma Corydalis	Release pain
Ge-gen	Radix Puerariae	Releases the neck and upper back muscles Discharges measles Anti-inflammation
Huang-Qin	Radix Scutellariae	Anti-inflammation Anti-tumor
Dan-Shen	Salviae Miltiorrhizae Radix et Rhizoma	Improve micro-circulaiton anit-inflammation
Mai-Dong	Ophiopogonis Radix	Treat for dry mouth
Bai-Zhi	Radix Angelicae Dahuricae	Anti-inflammation and anti-cancer
Pu-Gong-Ying	Taraxaci Herba	Anti-inflammation and reduces abscesses
Xiang-Fu	Rhizoma Cyperi	Releases pain and regulates menstruation
Hai-Piao-Xiao	Sepiae Endoconcha	Anti- stomach acid and release epigastric pain
Bai-Hua-She-She-Cao	Hedyotidis Herba	Clears Heat and reduces abscesses Anti-inflammation, anti-cancer
**Multi-herb products**		
Jia-Wei-Xiao-Yao-San	Radix Angelicae Sinensis, Radix Paeoniae Alba, Poria, Radix Angelicae Dahuricae, Radix Bupleuri, Cortex Moutan, Gardenia Glycerite, Radix Glycyrrhizae Preparata, Menthae Haplocalycis Herba, Rhizoma Zingiberis Recens	Treat for anxiety and release stress Release menstrual pain
Shu-Jing-Huo-Xue-Tang	Radix Paeoniae Alba, Radix Angelicae Sinensis, Rhizoma Chuanxiong, Radix Rehmanniae, Semen Persicae, Rhizoma Atractylodis, Poria, Radix Achyranthis Bidentatae, Radix Clematidis, Radix Stephaniae Tetrandrae, Rhizoma et Radix Notopterygii, Radix Saposhnikoviae, Radix Gentianae, Radix Angelicae Dahuricae, Pericarpium Citri Reticulatae, Radix Glycyrrhizae, Rhizoma Zingiberis Recens	Treat for neuralgia, and lumbago
Shao-Yao-Gan-Cao-Tang	Radix Paeoniae Alba, Radix Glycyrrhizae	Muscle relaxation
Yin-Qiao-San	Flos Lonicerae, Fructus Forsythiae, Radix Platycodi, Fructus Arctii, Herba Menthae Haplocalycis, Semen Sojae Preparatum, Herba Lophatheri, Herba Schizonepetae, Rhizoma Phragmitis, Radix Glycyrrhizae	Anti-common cold, anti-virus, treat for acute tonsillitis and fever
Gan-Lu-Yin	Radix Rehmanniae, Radix Rehmanniae Preparata, Herba Dendrobii, Radix Asparagi, Radix Ophiopogonis, Radix Scutellariae, Herba Artemisiae Scopariae, Fructus Aurantii, Folium Eriobotryae, Radix Glycyrrhizae	Treat for tomatitis and radiotherapy effects for breast cancer
Xiang-Sha-Liu-Jun-Zi-Tang	Radix Ginseng, Rhizoma Atractylodis Macrocephalae, Poria, Radix Glycyrrhizae, Pericarpium Citri Reticulatae, Rhizoma Pinelliae Preparatum, Fructus Amomi, Radix Aucklandiae	Treat for indigestion and for peptic ulcer and gastroenteritis
Ping-Wei-San	Rhizoma Atractylodis, Cortex Magnoliae Officinalis, Pericarpium Citri Reticulatae, Radix Glycyrrhizae, Rhizoma Zingiberis Recens, Fructus Jujube	Treat for peptic ulcer and gastroenteritis Anti- gastric cancer
Ban-Xia-Xie-Xin-Tang	Rhizoma Pinelliae Preparatum, Rhizoma Zingiberis, Radix Scutellariae, Rhizoma Coptidis, Radix Ginseng, Fructus Jujube, Radix Glycyrrhizae	Treat for peptic ulcer and gastroenteritis Anti- gastric cancer
Chai-Hu-Shu-Gan-Tang	Pericarpium Citri Reticulatae, Radix Bupleuri, Rhizoma Chuanxiong, Fructus Aurantii, Radix Paeoniae Alba, Radix Glycyrrhizae, Rhizoma Cyperi	Release abdominal pain Anti-gastric cancer and liver cancer Release breast distention
Suan-Zao-Ren-Tang	Semen Zizyphi Spinosae, Poria, Rhizoma Anemarrhenae, Rhizoma Chuanxiong, Radix Glycyrrhizae	Treat for insomnia and stress

## Discussion

In this evidence-based cohort study exploring the relationship between CHM use and subsequent depression risk in breast cancer patients, we found that patients receiving CHM had a nearly 25% lower risk of depression than did those not using CHM. Furthermore, those receiving CHM for more than 365 days were found to have a significantly lower risk of depression by 43%. Through the determination of dose-response relation between an exposure citation(CHM use) and the risk of an outcome citation(depression incident), it may indeed support the causal association between the intensity of CHM use and the resultant lower risk for depression among breast cancer patients.

No previous studies have reported the long-term influence of CHM on depression risk in breast cancer patients, thus precluding comparison of results. Nevertheless, the positive therapeutic effect of CHM observed in this work adds to the accumulating evidence suggesting that CHM exerts antidepressant effects by modulating dysfunctional neurotransmission (So et al., 2015). Proposed mechanisms by which CHM use might decrease the risk of developing depression include the inhibition of the monoamine oxidase activity and oxidative stress, upregulation of neurotrophins, and modulation of hypothalamic-pituitary-adrenal axis function (Dowlati et al., 2010; Wang Y. et al., 2019).

A major contribution of this study is the identification of specific CHM products associated with the observed lower risk of depression in breast cancer patients. We found that three single-product CHM commonly used to treat breast cancer were associated with a lower sequent risk of depression, like use of Ge-gen, Xiang-fu, and Bai-hua-she-she-cao. A previous study using an animal model reports that Pueraria, the principal ingredient of Ge-gen, exerted marked anti-depressant effects by abating nuclear factor kappa beta (NF-κB) signaling (Chang et al., 2009). NF-κB is a critical transcriptional regulator of inflammatory response induction (Liu T. et al., 2017), and a decrease in such signaling thereby decreases the vulnerability to depression.

Xiang-fu is a cytotoxic herb that inhibits cell growth and promotes apoptosis of breast cancer cells (Park et al., 2014; Mannarreddy et al., 2017; Simorangkir et al., 2019; Suryavanshi et al., 2019; Wang F. et al., 2019). A recent *in vivo* study by Zhou et al. (2016) showed that two new cycloartane glycosides from the rhizomes of Xiang-fu were beneficial in modulating the function of brain-derived neurotrophic factor in the hippocampus, a compound known to promote synaptic efficacy, neuronal connectivity, and neuroplasticity, which in turn decrease the risk of depression.

Another commonly used single-herb product, Bai-Hua-She-She-Cao (Hedyotis diffusa), is commonly used to relieve heat and toxicity in cancer patients (Zhang et al., 2017). Recent animal experiments and human studies have shown that the administration of Bai-Hua-She-She-Cao may markedly suppress cancer cell proliferation and induced apoptosis by modulating pro-inflammatory cytokines (Ye et al., 2015; Zhang et al., 2017), which may account for its therapeutic effect of preventing the onset of psychiatric disorders, including depression.

With regard to multi-herb products, the use of Shao-Yao-Gan-Cao-Tang was observed to be associated with a decreased risk of depression in our cohort. A previous study reported that an active component of Shao-Yao-Gan-Cao-Tang decoction, such as glycyrrhizin, affected multiple biological processes and pathways that participate in neuron apoptosis and inflammatory responses (Chen et al., 2018), all of which play key roles in the pathogenesis of depression (Dowlati et al., 2010). Another commonly used CHM product, Yin-Qiao-San, is used to relieve external symptoms and clear pathogenic heat from the body in clinical practice. This herbal product was proven to prevent drug-induced pulmonary fibrosis in a mice model by decreasing the plasma levels of inflammatory cytokines, like TNF-α, interleukin citation(IL)-1, and IL-6 (Yeh et al., 2007); these mediators have been implicated in the development of depression (Dowlati et al., 2010).

As previously described in “Materials and methods” section, the database used in this study has several strengths, including its large sample size, the use of electronic records from a national health insurance registry, and a uniform approach to outcome assessment, all of which could strengthen validity of the study findings. On top of that, the selection bias ought to be minimized to a great extent by using data from the NHI database, which included over 99% of insured persons in Taiwan. Nonetheless, several limitations of this study merit attention. First, our findings were derived from analysis of retrospective cohort data based on ICD-9-CM diagnostic codes. Thus, relevant cases may have been misclassified. To improve diagnostic accuracy and avoid overestimation of the prevalence of cases of interests, we applied a 4-year washout period for breast cancer, where patients with a diagnosis of breast cancer during that period were considered prevalent and, therefore, excluded from analyses. Medical records were confirmed by requiring at least 3 outpatient service claims, or at least 1 inpatient hospitalization claim during the study period. Additionally, the diagnostic inclusion of breast cancer was further verified by the catastrophi illness certification. The NHI of Taiwan randomly reviews the charts and audits medical charges to verify the accuracy of claims. Despite this procedure, we recognize that the misclassification of cases is always a risk in database research, but this would be expected to occur at random which would likely serve to only bias the overall HR observed toward the null value and thus provide a more conservative estimate of effect. Second, data used in this study were drawn from a claim-based database and, accordingly, no detailed clinical information regarding laboratory data, family history, physical activity, and individual social network relationship was available. Thus, future studies that examine the influence of these variables are worthy of pursuit. Nevertheless, to carefully address the confounding by indication that may arise in this real-world comparative effectiveness study, we have capitalized on propensity score—matched analysis to analyze the association of CHM use with the attack of depression. For each breast cancer patient who received CHM, one control patient was selected by 1:1 matching, based on a propensity score. Additionally, to more robustly assess if other treatments may affect the finding herein, we reanalyzed the relationships between CHM use and subsequent risk of depression by adjusting for the utilization numbers of radiotherapy after breast cancer onset. The rational underlying this procedure was based on the assumption that radiotherapy for breast cancer patients is an obligatory component of a breast-conserving treatment modality and could cause deleterious psychological symptoms in such a group of patients (Almasri and Rimawi, 2020; Piroth et al., 2022). The re-analysis indicated that, as compared to breast cancer patients who did not receive CHM, the selected breast cancer patients who received CHM still had a significantly lower risk for depression (adjusted HR: 0.75; 95% CI = 0.59–0.89). Third, other indicators of comorbidities, aside from CCI score, deserve further investigation while examining the association of depression with CHM use, among such group. Fourth, a surveillance bias might arise with this type of exploration, as CHM users may be more likely than their control counterparts to seek medical care. To address this issue, we calculated the frequency of medical visits for each study subject and adjusted this variable in the multivariate regression model. After adjustment, findings from the reanalysis indicated that the positive effect of CHM still remained unchanged, with an adjusted HR of 0.79 (95% CI = 0.62–0.96), suggesting that the number of ambulatory care visits did not appreciably affect the conclusion herein. Fifth, due to the anonymity of identification numbers in the database, we were unable to directly contact the enrolled patients regarding their methods of taking CHM products. However, nearly 95% of the dose frequencies in Chinese herbal products are typically used for only 1 week in clinical practice, so those who continued to receive the same prescription for a longer period of time were, therefore, likely to have used the prescribed medications (Lai et al., 2010). Additionally, prescriptions for medications issued before NHI initiation were not reflected in the data analysis in the present study. This omission may result in underestimating the cumulative frequencies and may have weakened the effect of the specified CHM products. Last, although our study revealed a substantial association between CHM use and a decreased risk of depression among breast cancer patients, it must be recognized that participants were not initially randomly categorized into users and non-users and were recruited from a single country only. Therefore, a randomized controlled trial to test the effects of those commonly used herbal products in treating breast cancer patients is warranted, thus paving the way for further personalized therapies and clinical trials on the use of adjunctive therapy for the management of other types of malignancies.

## Conclusion

Individuals with breast cancer suffer from the mood disorders more frequently than the general population, especially depression. This is the first population-based survey that rigorously evaluated the impact of CHM use on the prevention of depression among them. We found that integration of CHM into the conventional treatment regimen for breast cancer for more than 2 years may reduce the subsequent risk of depression by nearly 50%. This noteworthy finding can provide an impetus for further research venues, such as focusing on factors associated with utilization of CHM, as well as *in vivo* studies that attempt to uncover the main mechanisms of prescribed herbal formulae that may be beneficial for such groups.

## Data availability statement

The data supporting the conclusion of this study are available from the authors, but the raw data (NHIRD) needs to be obtained from the National Health Research Institute of Taiwan through an application process upon approval, further inquiries should be directed to the corresponding author.

## Ethics statement

This study was approved by the Research of Ethics Committee of Dalin Tzuchi Hospital. Written informed consent for participation was not required for this study in accordance with the national legislation and the institutional requirements.

## Author contributions

SY-Y, C-CY, C-KW, and T-YT: conception and design. M-CL and T-YT: provision of study material and patients. HL and T-YT: collection and assembly of data. SY-Y, HL, JS-J, S-WY, C-KW, and T-YT: data analysis and interpretation. SY-Y, HL, JS-J, S-WY, H-LH, MC, C-CY, C-KW, and T-YT: manuscript writing. SY-Y, HL, JS-J, S-WY, H-LH, MC, M-CL, C-CY, C-KW, and T-YT: final approval of manuscript. All authors contributed to the article and approved the submitted version.
